# Validity of Six Activity Monitors in Chronic Obstructive Pulmonary Disease: A Comparison with Indirect Calorimetry

**DOI:** 10.1371/journal.pone.0039198

**Published:** 2012-06-20

**Authors:** Hans Van Remoortel, Yogini Raste, Zafeiris Louvaris, Santiago Giavedoni, Chris Burtin, Daniel Langer, Frederick Wilson, Roberto Rabinovich, Ioannis Vogiatzis, Nicholas S. Hopkinson, Thierry Troosters, on behalf of PROactive consortium

**Affiliations:** 1 Faculty of Kinesiology and Rehabilitation Sciences, Department of Rehabilitation Sciences, Katholieke Universiteit Leuven, and Respiratory Division, UZ Gasthuisberg, Leuven, Belgium; 2 NIHR Respiratory Biomedical Research Unit at Royal Brompton and Harefield NHS Foundation Trust and Imperial College, London, United Kingdom; 3 Thorax Foundation, Research Centre of Intensive and Emergency Thoracic Medicine, and Department of Physical Education and Sports Sciences, National and Kapodistrian University of Athens, Athens, Greece; 4 ELEGI Colt Laboratory, Centre for Inflammation Research, University of Edinburgh, Edinburgh, Scotland, United Kingdom; 5 Precision Medicine, Pfizer Worldwide Research and Development, Sandwich, Kent, United Kingdom; University Hospital Freiburg, Germany

## Abstract

Reduced physical activity is an important feature of Chronic Obstructive Pulmonary Disease (COPD). Various activity monitors are available but their validity is poorly established. The aim was to evaluate the validity of six monitors in patients with COPD. We hypothesized triaxial monitors to be more valid compared to uniaxial monitors. Thirty-nine patients (age 68±7years, FEV_1_ 54±18%predicted) performed a one-hour standardized activity protocol. Patients wore 6 monitors (Kenz Lifecorder (Kenz), Actiwatch, RT3, Actigraph GT3X (Actigraph), Dynaport MiniMod (MiniMod), and SenseWear Armband (SenseWear)) as well as a portable metabolic system (Oxycon Mobile). Validity was evaluated by correlation analysis between indirect calorimetry (VO_2_) and the monitor outputs: Metabolic Equivalent of Task [METs] (SenseWear, MiniMod), activity counts (Actiwatch), vector magnitude units (Actigraph, RT3) and arbitrary units (Kenz) over the whole protocol and slow versus fast walking. Minute-by-minute correlations were highest for the MiniMod (r = 0.82), Actigraph (r = 0.79), SenseWear (r = 0.73) and RT3 (r = 0.73). Over the whole protocol, the mean correlations were best for the SenseWear (r = 0.76), Kenz (r = 0.52), Actigraph (r = 0.49) and MiniMod (r = 0.45). The MiniMod (r = 0.94) and Actigraph (r = 0.88) performed better in detecting different walking speeds. The Dynaport MiniMod, Actigraph GT3X and SenseWear Armband (all triaxial monitors) are the most valid monitors during standardized physical activities. The Dynaport MiniMod and Actigraph GT3X discriminate best between different walking speeds.

## Introduction

Chronic Obstructive Pulmonary Disease (COPD) is a chronic disease characterized by poorly reversible airflow limitation and destruction of lung parenchyma. However, COPD is now recognized as a systemic illness with significant extra-pulmonary features such as muscle wasting and weakness [1]. Physical inactivity is known to contribute to these extra-pulmonary features [2], [3]. A recent systematic literature review showed that physical activity is reduced in patients with COPD [4].

Physical activity is defined as any bodily movement produced by the contraction of skeletal muscle that increases energy expenditure above a basal level [5]. In the general population, lack of physical activity is associated with the burden of chronic disease. Similarly, there is increasing evidence that reduced physical activity worsens the prognosis of patients with COPD. Hence, inactivity is not only a manifestation of disease severity in COPD, but is intrinsic to disease progression [6].

Physical activity monitors are frequently used to estimate levels of daily physical activity. These devices use piezoelectric accelerometers, which measure the body’s acceleration, in one, two or three axes (uniaxial, biaxial or triaxial activity monitors). The signal can then be transformed into an estimate of energy expenditure using one of a variety of algorithms, or summarized as activity counts or vector magnitude units (reflecting acceleration). With the information obtained in the vertical plane or through pattern recognition, steps or walking time can also be derived by some monitors. The availability of sophisticated physical activity monitors has made the objective measurement of physical activity in COPD patients possible in a number of contexts, including assessment of the response to pharmacotherapy [7], during rehabilitation programmes [8] and during inpatient admission [9]. Most of the monitors currently available have been validated in healthy subjects, but not necessarily in patients with chronic diseases. As such patients are less physically active and move more slowly than healthy subjects [10], [11], the validity of these monitors to detect movement in these patients needs to be evaluated.

The aim of this study was to validate six physical activity monitors in COPD patients, against a gold standard of indirect calorimetry in the form of VO_2_ data from a portable metabolic system. Since triaxial accelerometers have previously been reported to be more effective compared to uniaxial accelerometers [12], we hypothesized that triaxial activity monitors would be more valid tools compared to uniaxial activity monitors. The work described here forms part of the EU/IMI-funded PROactive project to develop and validate a patient reported outcome for physical activity in COPD (www.proactivecopd.com).

## Materials and Methods

### Study Subjects

Ten patients were recruited in each of the 4 centres (Athens, Edinburgh, Leuven and London) to give a total number of 40 patients. All patients were diagnosed with COPD ranging in severity from mild to very severe according to the Global Initiative for Chronic Obstructive Lung Disease (or GOLD) (stages I to IV) [13]. They were clinically stable and free of exacerbations for at least 4 weeks prior to the study. Patients were excluded if they had other co-morbidities which would interfere with their movement patterns (e.g. arthritis), or if they were on long-term or ambulatory oxygen therapy, as they could not have supplemental oxygen whilst wearing the metabolic equipment. The protocol was approved by the ethics committee of each centre; Medical Ethical Board of the University Hospitals Leuven (Leuven, Belgium), NRES Committee London - Bloomsbury (London, United Kingdom), Sotiria Hospital Scientific and Ethics Commitee (Athens, Greece) and Lothian Regional Ethics Committee (Edinburgh, United Kingdom). Patients provided written informed consent.

### Pulmonary Function Testing

All pulmonary function measurements were performed with standardized equipment and according to American Thoracic Society and European Respiratory Society guidelines [14]. Post-bronchodilator spirometry was measured and lung diffusion capacity was determined by the single breath carbon monoxide gas transfer method (DLCO). All variables are given as absolute values and expressed as percentages of the predicted reference values [15], [16].

### Six-minute Walking Test

Functional exercise capacity was determined by six minute walking distance (6MWD) [17]. Values were related to previously published reference values [18].

### Incremental Exercise Testing

A symptom-limited incremental cycle ergometer test according to the ATS/ACCP statement on cardiopulmonary exercise testing [19], was used to assess the maximal exercise capacity (peak VO_2_). The values of peak oxygen consumption were related to previously described reference values [20].

### COPD-specific Health-related Quality of Life Questionnaires

The St.-George’s Respiratory Questionnaire (SGRQ) provides a total score and three component scores for symptoms, activity and impacts. Each score ranges from 0 (no impairment) to 100 (worst possible) [21].

The COPD Assessment Test (CAT) covers eight items (cough, phlegm, chest tightness, breathlessness, going up hills/stairs, activity limitations at home, confidence leaving home, sleep and energy). Each item is scored from 0 to 5 giving a total score range from 0 to 40, corresponding to the best and worst health status in patients with COPD, respectively [22].

The Medical Research Council (MRC) dyspnoea scale rates the type and magnitude of dyspnoea according to five grades of increasing severity [23].

### Study Design

Each patient wore 6 activity monitors simultaneously which were selected as a result of a systematic review of the literature. These were two uniaxial activity monitors [Kenz Lifecorder (Kenz), Actiwatch (Actiwatch)], three triaxial activity monitors [RT3, Actigraph GT3X (Actigraph), DynaPort MiniMod (MiniMod)] and one multisensor activity monitor combining a triaxial accelerometer with different sensors [SenseWear Armband (SenseWear)]. More details about software, type, body location and outputs of these monitors can be found in **Table 1**.

**Table 1:** Details of type, location and output of the six activity monitors.

Name, Manufacturer (software)	Type	Location	Measured output	Estimated output
Kenz Lifecorder Plus Suzuken CoLtd., Nagoya, Japan (Physical ActivityAnalysis Software)	Uniaxial accelerometer	Waist (left)	Steps, activity score	EE, activity intensity level
Actiwatch, MiniMitterCo,Sunriver,OR, USA (Respironics Actiware 5)	Uniaxial accelerometer	Wrist (left)	AC	
RT3, Stayhealthy Inc. Monrovia, CA,USA (Stayhealthy RT3 Assist Version1.0.7)	Triaxial accelerometer	Waist (right)	AC, VMU	EE
Actigraph GT3X, Actigraph LLCPensacola, FL (Actilife 5)	Triaxial accelerometer	Waist (right)	Steps, AC	EE, activity intensity level
DynaPort® MiniMod, McRoberts BV,The Hague, The Netherlands	Triaxial accelerometer	Waist (lower back)	Steps, movement Intensity,different body positions	EE
SenseWear Armband, Bodymedia,Pittsburgh, PA, USA (SenseWearProfessional 6.0)	Multisensor device: triaxialaccelerometer + sensors (heatflux, galvanic skin response andskin temperature)	Upper left arm at triceps	Steps, activity intensity level	EE

AC; activity counts, VMU: vector magnitude unit, EE; energy expenditure.

**Figure 1:**
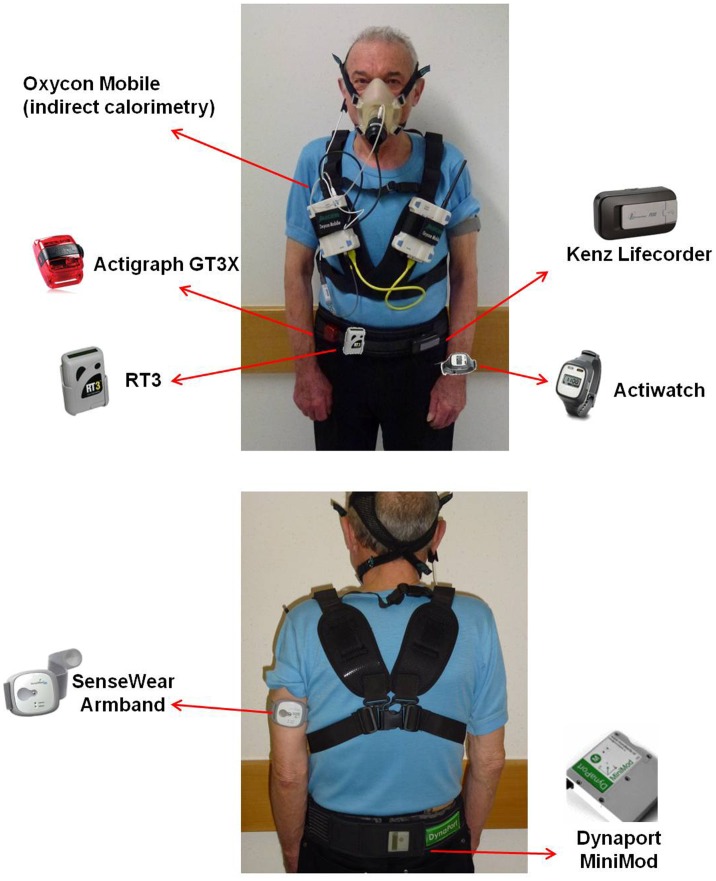
Location of attachment for the Oxycon Mobile and the six activity monitors.

Patients also wore a portable metabolic system (Jaeger Oxycon Mobile), an oxygen saturation finger probe and a Polar T31 (Polar) coded transmitter belt for heart rate monitoring. The portable metabolic system was attached to the upper chest with a harness and due to its low weight (950 g), caused minimal discomfort. A face mask with a dead space of <30 mL (Hans Rudolph Inc, Kansas City MO/USA) was used. Location of attachment for the Oxycon Mobile together with the six activity monitors is shown in **Figure 1**. A two-point gas calibration was completed prior to each test. Oxygen consumption (VO_2_), carbon dioxide production (VCO_2_), heart rate, respiratory rate and tidal volume were measured continuously. Breath-by-breath measurements were averaged over one-minute intervals. After the experiment, stored data were downloaded from the portable metabolic device to a personal computer. VO_2_ values were divided by participants’ body weight and converted to Metabolic Equivalents of Task (METs) [24]. Energy expenditure estimates from the portable metabolic system (METs) were used as a criterion measure for energy expenditure and were compared with the following activity monitor outputs: Kenz - arbitrary units (AU); Actiwatch - activity counts (AC); Actigraph and RT3 - vector magnitude units (VMU); MiniMod and SenseWear - METs.

Patients were instructed to perform a strict schedule of activities lasting 59 minutes (**Table 2**) which were chosen to be representative of everyday tasks (such as walking, stair climbing and sweeping the floor) that are reported as problematic by COPD patients [25]. Time was kept with both a stopwatch and a laptop computer clock so that activities were completed in whole minutes.

**Table 2:** Schedule of physical activities in the standardized protocol.

Activity	Duration (minutes)
Standing	1
Lying	3
Sitting	2
Standing	2
Slow walk*	6
Sitting	2
Standing	2
Fast walk*	6
Sitting	2
Standing	2
Sweeping	2
Sitting	2
Standing	2
Lifting	2
Sitting	2
Walking/standing	1
Stairs	1
Sitting	5
Walking/standing	1
Walking on treadmill (flat)**	4
Standing	2
Walking on treadmill (4% incline)**	4
Walking/standing	1
Sitting	2

* These walking activities were performed in a 30 m corridor. Speeds were self-selected. During fast walking, patients were instructed to walk as fast as possible.

** Participants walked at 85% of their fast walking speed, first on the flat and then at an incline of 4%. Participants were instructed not to support their arms during treadmill walking.

### Statistical Analysis

Minute-by-minute data from all devices were compiled for each patient in one database and synchronisation was verified by inspection of the curves to ensure the best fit between the monitors on a patient-by-patient basis. Analyses were carried out as follows (Pearson Product Moment Correlation was used for all correlation analyses):

1. A minute-by-minute correlation between METs from the portable metabolic system and each of the activity monitor outputs was calculated for every patient. Correlations between minute-by-minute VO_2_ and activity monitor outputs were reported as median with interquartile range. A Kruskal-Wallis test was used to compare results between different activity monitors. A median correlation larger than 0.7 was defined *a priori* as representing evidence of validity.

To investigate whether correlations became weaker with decreasing six minute walking distance (i.e. slower overall walking speed), and therefore if a monitor’s performance worsened as patients moved more slowly, the relationship between these minute-by-minute correlations and the six-minute walking distance (6 MWD) was tested. Correlation coefficients (minute-by-minute) in patients with mild to moderate COPD (GOLD I/II) were compared to those with severe to very severe COPD (GOLD III/IV).

Statistical analyses were performed with SAS software (version 9.2). A p-value <0.05 was considered to be statistically significant. Figure construction was performed with GraphPad Prism Version 4.0.

## Results

Patient characteristics are listed in **Table 3**. One patient was excluded from the analysis due to a technical problem with the collection of breath-by-breath data, leaving 39 patients in the final analysis. Due to technical problems, data collected from the RT3 in one centre could not be used, leaving 29 patients with RT3 in the final analysis. None of the included patients reported significant co-morbidities and all had normal exercise electrocardiograms.

**Table 3:** Characteristics of the 39 patients.

Variable	COPD patients (n = 39)
Age (years)	67.9±7.4
Gender (male/female, n)	25/14
FEV_1_ (L)	1.43±0.60
FEV_1_ (%predicted)	54±18
FVC (L)	2.97±0.85
FVC (%predicted)	90±16
GOLD stage I/II/III/IV (n)	4/18/14/3
BMI	26.2±5.2
6 MWD (m)	438±115
6 MWD (%predicted)	70±18
VO_2peak_ (ml*min^−1^*kg^−1^)	16.9±5.5
VO_2peak_ (%predicted)	79±31
MRC	2.6±0.7
CAT	15±8
SGRQ	
Total Score	42±18
Activities	60±24
Impacts	30±17
Symptoms	44±23

Data are expressed as mean ± std. FEV_1_; forced expiratory volume in 1 s, FVC; forced vital capacity, 6 MWD; six-minute walking distance, MRC; Medical Research Council, CAT; COPD Assessment Test, SGRQ; St George’s Respiratory Questionnaire.

An example of one patient’s data is provided **Figure S1**. The mean VO_2_ for all activities during the experiment was 8.5±1.5 ml*min^−1^*kg^−1^ which corresponded to a moderate intensity of activity (i.e. approximately 50% of VO_2peak_).

**Figure 2:**
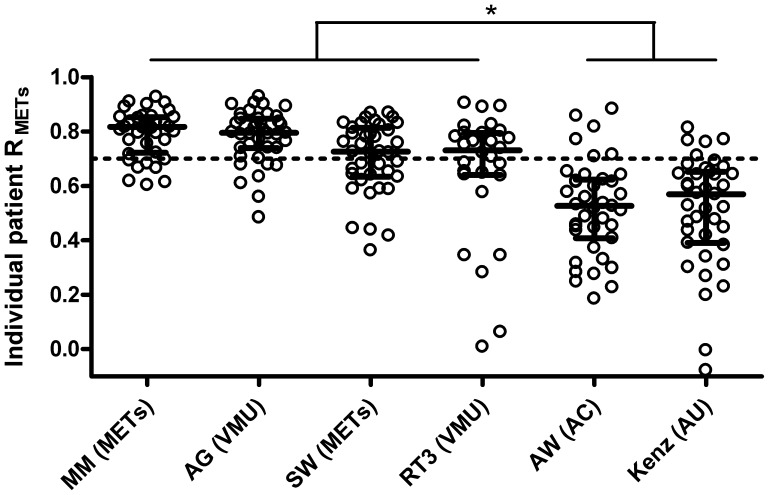
Minute-by-minute correlations (R) between activity monitor outputs and metabolic equivalents of task (METs) per patient (white dots). MM; MiniMod, AG; Actigraph, SW; SenseWear, AW; Actiwatch, VMU; vector magnitude unit, AC, activity count, AU; arbitrary unit. Dotted line corresponds to a correlation of 0.7, defined *a priori* as supporting monitor validity. Median (interquartile range) correlation for each activity monitor is reflected by cross bars, *p<0.05.

Minute-by-minute correlations between metabolic cost (METs) and activity monitor output are shown in **Figure 2**. Strong correlations (R>0.7 [interquartile range, IQR]) were found with the MiniMod (0.82 [IQR 0.72 to 0.85]), Actigraph (0.79 [IQR 0.74 to 0.85]), SenseWear (0.73 [IQR 0.63 to 0.81]) and RT3 (0.73 [IQR 0.64 to 0.79]) compared to the Actiwatch (0.53 [IQR 0.41 to 0.62]) and Kenz (0.57 [IQR 0.39 to 0.65]). This difference was also statistically significant (p<0.05).

These individual minute-by-minute correlations [95% confidence interval (95% CI)] were moderately but significantly related to the 6MWD for the Actiwatch (r = 0.60 [95% CI 0.35–0.77]), MiniMod (r = 0.51 [95% CI 0.23–0.71]), SenseWear (r = 0.48 [95% CI 0.19–0.69]) and Actigraph (r = 0.47 [95% CI 0.18–0.69]). No differences were observed for minute-by-minute correlations in mild to moderate COPD (GOLD I/II) compared to severe and very severe COPD (GOLD III/IV) as showed in **Table 4**.

**Table 4:** Minute-by-minute correlations between indirect calorimetry (METs) and activity monitor output in mild to moderate COPD (GOLD I/II) and severe to very severe COPD (GOLD III/IV).

Activity monitor output	GOLD I/II (n = 22)	GOLD III/IV (n = 17)
MiniMod (METs)	0.82 [0.81–0.86]	0.77 [0.68–0.83]
SenseWear (METs)	0.78 [0.68–0.83]	0.65 [0.59–0.75]
Actigraph (VMUs)	0.81 [0.74–0.86]	0.77 [0.74–0.83]
ActiWatch (Activity counts)	0.58 [0.46–0.64]	0.45 [0.28–0.53]
RT3 (VMUs)	0.69 [0.34–0.78]	0.76 [0.64–0.79]
Kenz (Arbitrary units)	0.54 [0.42–0.64]	0.59 [0.38–0.65]

Data are expressed as median [interquartile range]. METs; Metabolic Equivalents of Task, VMUs; Vector Magnitude Units.

**Figure 3:**
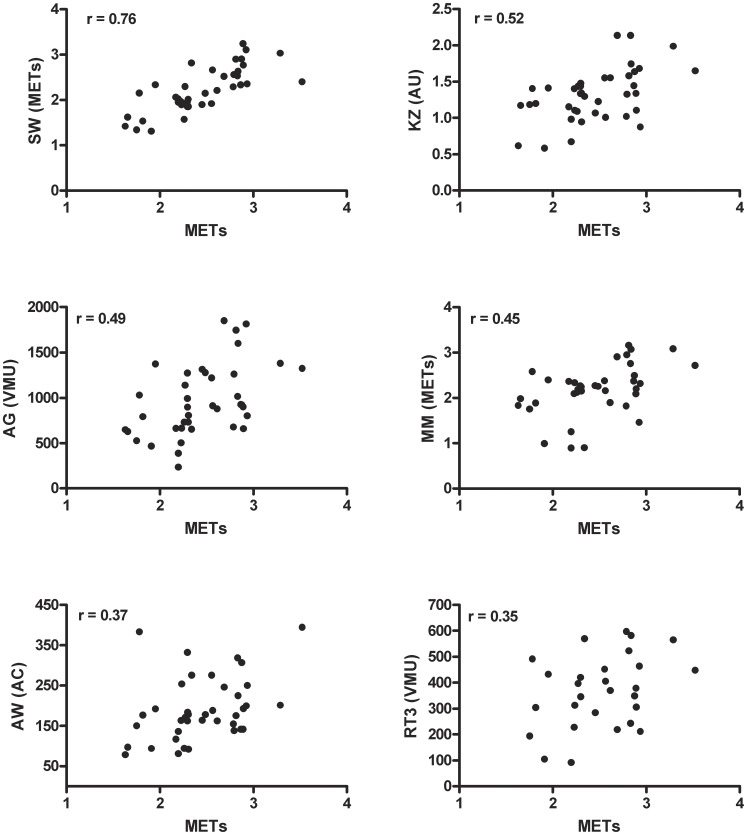
Relation between the activity monitor outputs and indirect calorimetry (METs). Data points represent mean values over the whole protocol. MM; MiniMod, AG; Actigraph, SW; SenseWear, AW; Actiwatch, VMU; vector magnitude unit, AC; activity count, AU; arbitrary unit.

The mean correlation between metabolic cost (METs) and activity monitor outputs over the whole protocol was, from highest to lowest; SenseWear (r = 0.76 [95% confidence interval (95% CI) 0.54–0.91]), Kenz (r = 0.52 [95% CI 0.27–0.73]), Actigraph (r = 0.49 [95% CI 0.28–0.64]), MiniMod (r = 0.45 [95% CI 0.21–0.61]), Actiwatch (r = 0.37 [95% CI 0.17–0.56]), all p<0.05 and RT3 (r = 0.35 [95% CI −0.04–0.48], p = 0.06) **(** **Figure 3** **)**.

**Figure 4:**
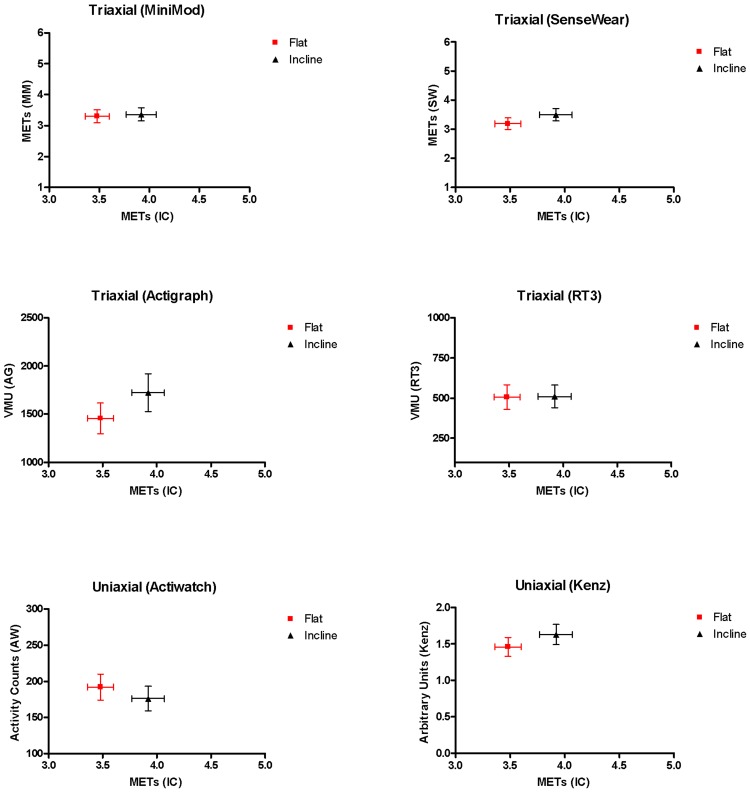
METs (derived from indirect calorimetry (IC)) and different activity monitor outputs during flat and inclined walking on a treadmill (both at the same speed (85% of their fastest walking speed during 6 MWT). MM; MiniMod, SW; SenseWear, AG; Actigraph, AW; Actiwatch. Symbols represent the mean, error bars the standard error of the mean.

Patients changed their walking speed by 1.31 km/h from the slow (3.27±0.47 km/h) to the fast (4.65±1.28 km/h) walking phase. As expected, the change in walking speed correlated with the change in VO_2_ determined by the metabolic equipment (r = 0.65) and rose from 3.11±0.74 METs to 3.93±1.23 METs. All monitors detected this increase in energy expenditure during fast walking compared to slow walking via their outputs. The highest correlations were reported for the MiniMod (r = 0.94 [95% confidence interval (CI) 0.89–0.97]) and Actigraph (r = 0.88 [95% CI 0.77–0.93]) compared to the RT3 (r = 0.69 [95% CI 0.42–0.85]), Actiwatch (r = 0.59 [95% CI 0.34–0.76]), Kenz (r = 0.57 [95% CI 0.31–0.76]) and SenseWear (r = 0.52 [95% CI 0.25–0.72]), all p<0.0001. As one would expect, walking on an incline on the treadmill (3.92±0.92 METs) expended more energy than walking on the flat (3.48±0.75 METs), as measured by indirect calorimetry (p<0.0001), but no differences between the two activities were detected by any of the activity monitors **(** **Figure 4** **)**.

**Figure 5:**
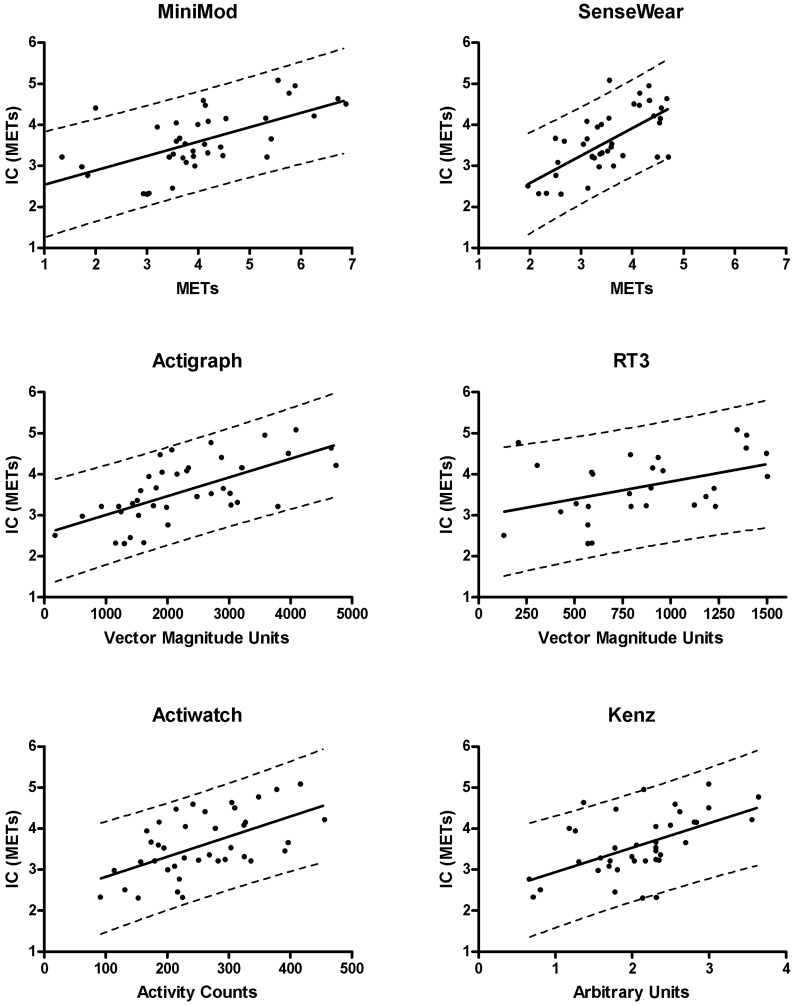
Bland regression analysis between METs derived from indirect calorimetry (IC) and different activity monitor outputs. Solid lines represent regression lines, dotted lines represent 95% limits of prediction.

Bland regression analyses demonstrated significant relationships (p<0.05) between METs derived from indirect calorimetry and the different activity monitor outputs, except for the RT3 (p = 0.06) **(** **Figure 5** **)**. The 95% limits of prediction for METs (derived from indirect calorimetry) at the mean (3.59 METs) were from lowest to highest: ±1.13 METs (SenseWear), ±1.15 METs (Actigraph), ±1.17 METs (MiniMod), ±1.25 METs (Actiwatch), ±1.28 METs (Kenz) and ±1.41 METs (RT3).

The Sensewear, Actigraph and MiniMod monitors together explained 62% of the variance in mean VO_2_. Partial variances by different sequences of activity monitors are presented in **Table 5**. Most variance in mean VO_2_ was explained by the SenseWear (58%) compared to the Actigraph (24%) and the MiniMod (21%). Little variance in mean VO_2_ was explained over and above the SenseWear, when it was introduced into the regression model first. In fact, introduction of the SenseWear further improved prediction models from other monitors (range 34 to 41%) compared to Actigraph (range 0 to 7%) and MiniMod (range 3 to 4%).

**Table 5:** Partial variance (pR^2^) of the activity monitors (AM) in the six linear regression models.

AM1 - AM2 - AM3(inserted in the model in this order)	pR^2^(AM1)	pR^2^(AM2)	pR^2^(AM3)
SenseWear - MiniMod - Actigraph	0.58*	0.04	0.00
SenseWear - Actigraph - MiniMod	0.58*	0.01	0.03
MiniMod - SenseWear - Actigraph	0.21	0.41*	0.00
MiniMod - Actigraph - SenseWear	0.21	0.07	0.34*
Actigraph - MiniMod - SenseWear	0.24	0.03	0.35*
Actigraph - SenseWear - MiniMod	0.24	0.35*	0.03

## Discussion

The validity of six commercially available activity monitors was investigated by comparing activity monitor outputs for each monitor to actual VO_2_ measured with indirect calorimetry. Triaxial activity monitors were judged to be more valid compared to uniaxial activity monitors according to a number of pre-defined criteria. Correlations between minute-by-minute outputs and VO_2_ were the highest with the Dynaport MiniMod, Actigraph GT3X, SenseWear Armband and RT3, all exceeding the *a priori* threshold of 0.7. Similarly the average monitor output over the 59 minute assessment was related to the average VO_2_ with the best correlations reported for three triaxial activity monitors (SenseWear Armband (r = 0.76), Actigraph GT3X (r = 0.49) and Dynaport MiniMod (r = 0.45)) and one uniaxial activity monitor (Kenz Lifecorder (r = 0.52)). All monitors were able to detect modest changes in walking speed but two triaxial activity monitors had the strongest correlations (MiniMod (r = 0.94) and SenseWear (r = 0.88)). Walking on an incline was more intense compared to flat walking when assessed with indirect calorimetry but no differences were detected by any of the monitors. All activity monitor outputs showed similar variability in predicting energy expenditure. The 95% prediction limits for mean METs (3.59 METs) varied between ±1.13 METs (SenseWear) and ±1.41 METs, (RT3). This implies that, from a clinical perspective, predicting oxygen consumption directly from different activity monitor outputs is not accurate. However, when patients engage in activity, monitors are highly capable of detecting the increase in physical activity levels within a range of 1 to 1.5 METs.

This is the first multi-center trial where several activity monitors have been validated against VO_2_ in different stages of COPD. Until now, only the Dynaport MiniMod and SenseWear Armband had been validated against VO_2_ in patients with COPD. In these devices, similar correlation coefficients were previously reported between activity monitor outputs and total energy expenditure (r = 0.75 and r = 0.93 for the SenseWear Armband [27], [28], r = 0.7 for the Dynaport Minimod [27]). During a set of 5 daily activities, a high level of agreement between SenseWear Armband (22.7±7 kcal) and indirect calorimetry (21.0±7.9 kcal) was observed [29]. In a similar protocol, a fair agreement between energy expenditure estimate from the SenseWear Armband and energy expenditure measure from indirect calorimetry was reported (mean difference of −0.2 METs with a limit of agreement of 1.3 METs) [30].

In the present study, validity was assessed using correlation analysis rather than a measure of agreement (e.g. Bland and Altman analysis), as most of the activity monitor outputs are in different units (activity counts, vector magnitude units, etc.) to each other, and to the VO_2_ data. It is not possible to convert all monitor outputs to energy expenditure. Several prediction equations are available to convert some outcomes (e.g. VMU) to energy expenditure and energy expenditure can also be derived from the VO_2_ data for direct comparison. However, whilst the prediction equations used by the Actigraph (7164 model and GT1M), Actiwatch, Kenz and RT3 are publicly available, [31], [32], [33], [34], the SenseWear and MiniMod use proprietary algorithms developed by the device manufacturers. Moreover, the goal of the study was to assess the validity of the devices rather than their prediction equations. Therefore, comparing the raw data from the activity monitor with the VO_2_ (derived from the portable metabolic kit) by using correlation analysis was the most appropriate statistical approach.

In addition, energy expenditure is driven to a large extent by a number of other factors. Whilst specific factors such as body weight, age, and height can be incorporated into prediction equations, it is more difficult to include non-specific factors such as mechanical efficiency, especially in patients with COPD. Patients with COPD have a larger active energy expenditure [35] even though it is well recognized that they are moving less. This is consistent with findings of reduced mechanical efficiency in these patients compared to healthy controls [36], [37]. An activity monitor cannot be expected to incorporate such a complex change in an estimate of energy expenditure, so perhaps greater weight should be placed upon direct monitor outputs (steps, activity counts, vector magnitude units, etc.). In essence, this means that activity monitors are most appropriately used for the assessment of the activities of patients in terms of amount and/or intensity and it should be acknowledged that the derivation of energy expenditure is imperfect. Therefore, the use of derived energy expenditure is also not appropriate when assessing monitor performance.

The ability of an activity monitor to pick up a difference in walking speed of 1.31 km/hr is clinically relevant. Patients with COPD do walk more slowly than healthy subjects, which is reflected, for example, by their reduced six minute walking distance [38], [39]. In our study, all monitors were able to detect these modest changes in walking speed.

A limitation of this study was that inter- and intra-device reliability of the different activity monitors was not evaluated. This is important when physical activity levels of patients are followed over time. Several studies have shown moderate to high inter-device reliability (intra-class correlation coefficient (r_ICC_) = 0.99 for the Actigraph Model 7164 [40], r_ICC_ = 0.95 for the Kenz Lifecorder [41] and r_ICC_ = 0.75 for the RT3 [42]) as well as intra-device reliability (r_ICC_ = 0.98 for the Actiwatch [43], r_ICC_ = 0.97 for the SenseWear Armband [44] and r_ICC_ = 0.86–0.99 for the Dynaport Minimod [45]). Besides the concepts of validity and reliability, other factors like size and scope of the study, usability and cost of the activity monitor need to be taken into consideration when selecting an activity monitor for use in clinical trials.

The validation of these activity monitors in a laboratory setting (validation against VO_2_) can be considered as an important step in ascertaining their validity. An essential next step will be to confirm their validity in a field setting.

In conclusion, this study found that three triaxial activity monitors (Dynaport MiniMod, Actigraph GT3X and SenseWear Armband) were the best monitors to assess standardized and common physical activities in the range of intensity relevant to patients with COPD. Changes in walking speed are most accurately registered by the Dynaport MiniMod and Actigraph, which are both devices that are worn on the hip. This should guide users in choosing valid activity monitors for research or for clinical use in patients with chronic diseases such as COPD.

## Supporting Information

Figure S1 **Example of one patient’s experiment; data of the Oxycon Mobile (VO_2_ (METs)) and the different activity monitor outputs.**
(TIF)
