# Supplementary figures and images for: Validity of Six Activity Monitors in Chronic Obstructive Pulmonary Disease: A Comparison with Indirect Calorimetry
Source: PLoS One. 2012 Jun 20;7(6):e39198. doi: 10.1371/journal.pone.0039198 (PMC3380044; doi:10.1371/journal.pone.0039198)

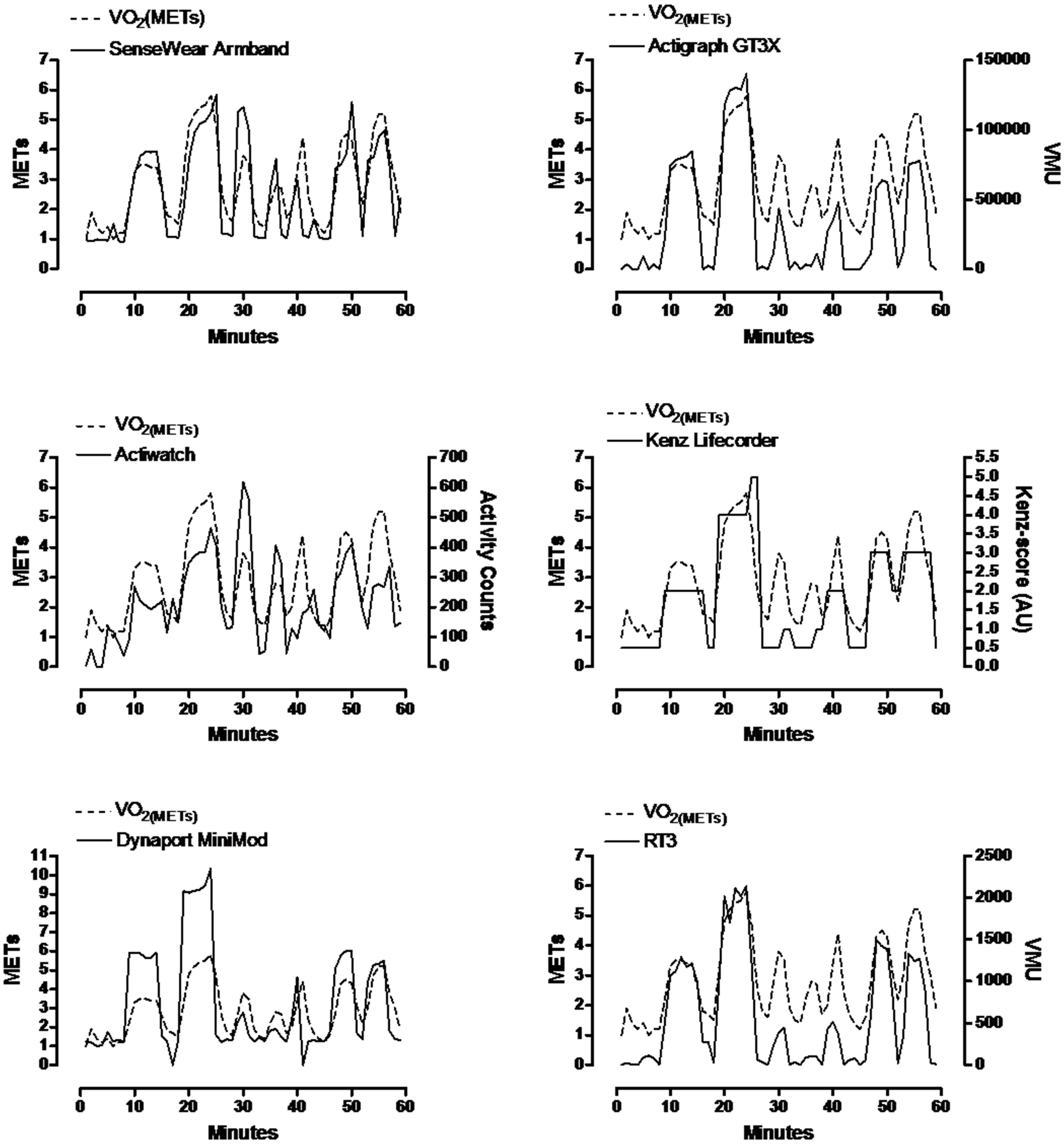

Supplement: Figure S1 — Example of one patient’s experiment; data of the Oxycon Mobile (VO2 (METs)) and the different activity monitor outputs. (TIF) [file pone.0039198.s001.tif]
